# Cooperative Control Strategy for Low-Voltage Ride-Through of DFIGM Based on an Improved IGBT-Based Active Crowbar

**DOI:** 10.3390/mi17020243

**Published:** 2026-02-13

**Authors:** Yu Zhang, Kai Li, Zhi Chen, Yutian Sun, Liangxing Hu

**Affiliations:** 1 School of Intelligent and Architectural Engineering, Harbin University, Harbin 150086, China; 2 College of Electrical Engineering, Harbin University of Science and Technology, Harbin 150080, China; 3 School of Electrical and Electronic Engineering, Nanyang Technological University, Singapore 639798, Singapore; 4 Harbin Electric Machinery Co., Ltd., Harbin 150040, China; 5 North Night Vision Technology Co., Ltd., Kunming 650217, China; 6 Kunming Institute of Physics, Kunming 650223, China

**Keywords:** doubly fed induction generator-motor (DFIGM), low-voltage ride-through (LVRT), improved active crowbar, current reversely tracking control (CRTC)

## Abstract

To address the low-voltage fault issue in doubly fed induction generator-motor (DFIGM) systems, this paper proposes a practically implementable cooperative control strategy that integrates an improved current reversely tracking control (CRTC) scheme with an enhanced IGBT-based active crowbar topology. The proposed method optimizes the current-tracking coefficients under rotor voltage and current constraints during LVRT operation. Meanwhile, the enhanced active crowbar provides reactive power support, thereby suppressing negative-sequence current components, mitigating harmonic distortion, and improving the power quality at the point of common coupling (PCC). A 10-MW DFIGM model is developed, and comparative studies are conducted with the conventional inductance emulating control (IEC) and the crowbar structure. The experimental results show the feasibility and effectiveness of the proposed method.

## 1. Introduction

Variable-speed pumped storage has a unique and wide range of power regulation capability, offering significant advantages in accommodating fluctuating and intermittent renewable energy. Therefore, it plays an important role in the construction of a strong and intelligent power grid [1]. However, owing to its special topology, DFIGM is fundamentally different from the conventional doubly fed induction generator (DFIG) employed in wind power applications. The DFIGM operates in both generating and motoring modes, which leads to more complex fault characteristics and protection requirements. Consequently, DFIGM is highly sensitive to grid disturbances. Without appropriate protective measures, grid faults may cause severe overcurrent in the rotor-side converter (RSC) and overvoltage on the DC-link, potentially resulting in converter damage [2].

The conventional LVRT control methods of DFIGM are mainly divided into two categories, one is based on the software control, the other is based on auxiliary devices [3]. A variety of software-based LVRT control strategies for DFIGM have been reported in the literature. In [4], a small-signal state-space model of the DFIG is established to investigate the influence mechanism of the phase-locked loop (PLL) on current control, and a composite decoupling control strategy considering the PLL dynamics is proposed. By analyzing the stability and unbalanced characteristics of positive- and negative-sequence rotor current control, coordinated compensation for unbalanced stator voltage and current is achieved in [5]. In [6], an inductance emulating control model is developed, which not only maximizes the cancelation of electromotive force (EMF) but also accelerates stator flux decay and suppresses torque ripple. On this basis, virtual capacitance is further introduced to propose an improved predictive current control scheme, which simplifies the flux measurement process [7]. In [8], an enhanced excitation converter is introduced by combining nonlinear passive control with genetic algorithms. Compared with the conventional PI controller, this method shows significant advantages in improving rotor current regulation capability; however, it fails to accelerate the attenuation of transient stator flux. In [9], a current-tracking control method is adopted, in which the stator current multiplied by a tracking coefficient is used as the reference value of rotor current feedback. At present, a wide variety of algorithmic models have been developed for the fault ride-through of DFIGM. However, when intelligent control methods are employed, the computational efficiency of optimization algorithms and the complexity of control structures must be carefully considered, which inevitably increases the difficulty of practical implementation. Moreover, due to inherent time delays, complex models fail to fully capture the entire process from voltage sag to steady-state recovery.

Relying solely on the capacity of RSC is insufficient to meet the requirements of LVRT. The crowbar branch control strategy is simple to implement and effective in suppressing rotor overcurrent. In recent years, extensive efforts have been devoted to improving the traditional crowbar scheme [10,11]. For instance, optimizing the control parameters of STATCOM has been reported to enhance the performance of DFIGs under fault conditions [12]. Other studies have realized the dynamic adjustment of crowbar resistance [13,14] and addressed the overmodulation problem of the DFIG converter by decoupling the RSC control loops [15]. By modifying the crowbar branch topology, equivalent resistance can be regulated through the duty cycle of IGBT switching. However, frequent resistance commutation tends to enlarge the fluctuation of the DC-link voltage around the threshold [16,17]. To further improve the power support capability of DFIGs during faults, various cooperative control strategies combining auxiliary hardware branches and intelligent methods have been proposed [18,19], including hybrid machine-learning-based control approaches [20]. In [21], the small-signal dynamic behavior of a DFIG under symmetrical faults in weak grids is investigated, where the interaction between the RSC and its control loops is analyzed, and the stability of the DFIG system is evaluated using the torque coefficient method. Moreover, an improved vector control strategy is proposed in [22,23], which is implemented in a single synchronous reference frame without decomposing negative-sequence voltages and currents, thereby simplifying the algorithm and providing a faster dynamic response. In summary, most crowbars still rely on the idea of a resistor to consume energy; the DFIGM still needs to absorb reactive power for excitation, which makes the power quality of the grid worse. The crowbar combined with power components optimizes the above problems, but the reactive power support capability is not improved fundamentally.

Following the analysis above, a cooperative control method is proposed in this paper; the main contributions can be summarized as follows:

(1) The scheme of CRTC method is developed, in which the current tracking coefficient is optimized based on particle swarm optimization (PSO) algorithm under rotor voltage and current constraints during LVRT conditions.
(2) An enhanced IGBT-based active crowbar topology is proposed, which operates in rectification and inversion modes and provides reactive power support, thereby improving fault ride-through capability.
(3) A cooperative control strategy integrating the optimized CRTC and the enhanced active crowbar is established, enabling effective mitigation of deep voltage sag faults, while suppressing the negative-sequence current components and harmonic content at the PCC.

The paper is organized as follows: In Section 2, the EMF and magnetic flux variation are analyzed. Section 3 demonstrates the cooperative control method. To verify the effectiveness of the proposed control method, a 10 MW DFIGM simulation model is established in Section 4; a similar experimental platform is set up in Section 5. Section 6 concludes this paper.

## 2. Characteristics of the DFIGM

In this section, the EMF and magnetic flux variation characteristics under normal operating conditions, as well as under symmetrical and asymmetrical voltage dips, are analyzed, respectively.

### 2.1. Normal Condition

**Figure 1:**
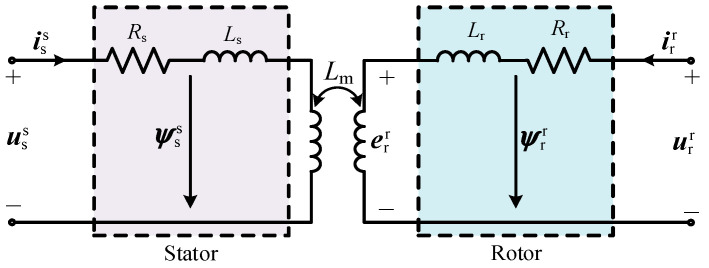
The equivalent circuit of DFIGM.

The stator and rotor windings of DFIGM are assumed to be Y-connected. The air-gap magnetic field is considered to be sinusoidally distributed; the effects of spatial harmonics, iron losses, and magnetic saturation are neglected. The machine parameters are constant and independent of frequency and temperature variations; the equivalent model is shown in Figure 1.

The DFIGM models in pumping and generating modes differ in the signs (±) of current-related variables. For clarity, the pumping mode is considered as a representative case, where the positive direction of the winding currents is defined as flowing into the machine.

(1) $$\left\{\begin{array}{l} u_{s}^{s}=R_{s}i_{s}^{s}+\frac{d}{dt}\psi_{s}^{s}=R_{s}i_{s}^{s}+\frac{d}{dt}(L_{s}i_{s}^{s}+L_{m}i_{r}^{s})\\ u_{r}^{r}=R_{r}i_{r}^{r}+\frac{d}{dt}\psi_{r}^{r}=R_{r}i_{r}^{r}+\frac{d}{dt}(L_{m}i_{s}^{r}+L_{r}i_{r}^{r}) \end{array}\right.$$

Among them, *L* and *R* denote inductance and resistance, respectively; *L*_m_ represents mutual inductance; *ψ*, *u*, and *i* are flux linkage, voltage and current vectors; the subscripts s and r represent the variables or parameters related to the stator and rotor, respectively; and the superscripts s and r represent that the vector has been converted to the stator and rotor sides.

The rotor voltage $u_{r}^{r}$ can also be expressed as shown in Equation (2), which consists of two parts: the EMF $e_{r}^{r}$ in rotor side and the voltage dip $u_{\mathrm{RL}}$ caused by rotor transient impedance.

(2) $$u_{r}^{r}=e_{r}^{r}+u_{\mathrm{RL}}=\frac{L_{m}}{L_{s}}\frac{d}{dt}\psi_{s}^{r}+(R_{r}+\sigma L_{r}\frac{d}{dt})i_{r}^{r}$$

σ is the leakage inductance coefficient and $\sigma L_{r}$ is the transient inductance of RSC.

### 2.2. Symmetrical Voltage Dip

Assuming that the symmetrical grid voltage dip occurs at *t* = 0 and the dip depth is *d*. there is the following expression of stator voltage $u_{s}^{s}$ in stator side:

(3) $$u_{s}^{s}=\left\{\begin{array}{l} U_{0}e^{j\omega_{s}t},\;t<0\\ (1-d)U_{0}e^{j\omega_{s}t},\;t\geq 0 \end{array}\right.$$

where *ω*_s_ is the synchronous angular frequency; *U*_0_ is the initial amplitude of stator voltage. When the grid voltage drops completely, the stator flux contains only the natural component. In the case of an incomplete voltage dip, the complete expression of the stator flux consists of the superposition of the forced component and the natural component.

(4) $$\psi_{s}^{s}=\psi_{\mathrm{sf}}^{s}+\psi_{\mathrm{sn}}^{s}=\frac{(1-d)U_{0}}{j\omega_{s}}e^{j\omega_{s}t}+\frac{dU_{0}}{j\omega_{s}}e^{-t/\tau_{s}}$$

where $\tau_{s}=L_{s}/R_{S}\tau_{S}$ is the stator time constant; both the components of the stator flux induce electromotive forces in the rotor, and their superposition yields the resultant EMF.

The sudden change in the grid voltage leads to the transient change of $e_{r}^{r}$. There are the forced component $e_{rf}^{r}$ and free component $e_{\mathrm{rn}}^{r}$ in $e_{r}^{r}$:

(5) $$e_{r}^{r}=\left\{\begin{array}{l} \frac{L_{m}}{L_{s}}U_{0}se^{j\omega_{r}t},\;t<0\\ e_{rf}^{r}+e_{m}^{r}=\frac{L_{m}}{L_{s}}U_{0}[s(1-d)e^{j\omega_{r}t}-(1-s)de^{-j\omega_{m}t}e^{-t/\tau_{s}}],\;t\geq 0 \end{array}\right.$$

where $e_{rf}^{r}$ rotates at the slip angular frequency ω_r_; its amplitude is very small, which is proportional to slip ratio *s*. $e_{\mathrm{rn}}^{r}$ rotates at the electrical angular frequency *ω*_m_ of the rotor; its initial amplitude is proportional to *d* and decays with $\tau_{s}$.

### 2.3. Asymmetrical Voltage Dip

Assuming that interphase short-circuit occurs between phase B and phase C, the fault will cause a large negative-sequence component; the zero sequence is not existent.

(6) $$\begin{array}{cc} \left\{\begin{array}{l} u_{s+}^{s}=(1-0.5d)U_{0}e^{j\omega_{s}t}\\ u_{s-}^{s}=0.5dU_{0}e^{j\omega_{s}t} \end{array}\right. & ,\;t\geq 0 \end{array}$$

Here, subscripts + and − represent positive and negative components, respectively. Compared with the symmetric dip, $e_{r}^{r}$ can be expressed as below:

(7) $$\begin{array}{c} e_{r}^{r}=e_{r+}^{r}+e_{r-}^{r}+e_{\mathrm{rn}}^{r}=\frac{L_{m}}{L_{s}}U_{0}[s(1-0.5d)e^{j\omega_{r}t}+0.5d(2-s)e^{-j(2-s)\omega_{s}t}\\ \;\;\;\;\;\;\;\;\;\;\;\;\;-(1-s)de^{-j\omega_{m}t}e^{-t/\tau_{s}}],\;t\geq 0 \end{array}$$

where one of $e_{r}^{r}$ is generated by $u_{s+}^{s}$, recorded as $e_{r+}^{r}$; the other is generated by $u_{s-}^{s}$, recorded as $e_{r-}^{r}$. $e_{r+}^{r}$ rotates at *ω*_r_; the amplitude of $e_{r+}^{r}$ is small, which is proportional to *s*. If *s* is small, the amplitude and frequency of $e_{r-}^{r}$ are multiplied by a coefficient of nearly 2, and its frequency is almost twice the synchronous frequency. If *d* is very large, the amplitude of $e_{r-}^{r}$ will be relatively large. $e_{\mathrm{rn}}^{r}$ is consistent with the symmetrical voltage dip.

## 3. Cooperative Control Method

**Figure 2:**
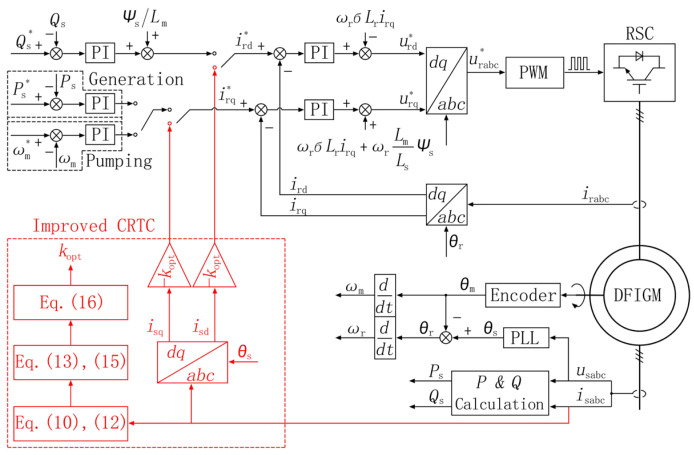
The control diagram of improved CRTC.

The working process of RSC is divided into generation mode and pumping mode. Under normal operating conditions, the vector control based on stator flux oriented is adopted in RSC, the control principle is shown in Figure 2. In this section, the proposed LVRT control scheme is introduced, the improved CRTC and crowbar are included.

### 3.1. Improved CRTC

If the dip depth is less than 50%, the DFIGM system can meet the requirements of LVRT by its own converter capacity. In this case, the CRTC method is put into operation, and the control performance of CRTC is optimized by PSO algorithm. The improved CRTC achieves a new balance between the suppression effects of rotor overcurrent and rotor overvoltage.

The stator flux will not change in a short time, no matter how $i_{s}^{r}$ and $i_{r}^{r}$ change; the vector sum of $i_{s}^{r}$ and $i_{r}^{r}$ is always equal to the stator magnetization current i_sm_:

(8) $$i_{\mathrm{sm}}=\frac{\psi_{s}^{r}}{L_{m}}=\frac{L_{s}i_{s}^{r}+L_{m}i_{r}^{r}}{L_{m}}=\frac{L_{s}}{L_{m}}i_{s}^{r}+i_{r}^{r}$$

Therefore, if the direction of $i_{r}^{r}$ is opposite to the stator current and smaller than the $i_{s}^{r}$, the rotor current can reduce the transient component in the stator flux linkage:

(9) $$i_{r}^{r*}=-ki_{s}^{r}$$

where *k* is tracking coefficient. Assuming that the rotor current can accurately track the current command, the rotor current can be solved when adopting CRTC:

(10) $$i_{r}^{r}=\frac{k}{k-L_{s}/L_{m}}i_{\mathrm{sm}}=\frac{k}{L_{m}k-L_{s}}\psi_{s}^{r}$$

The rotor voltage can be obtained as:

(11) $$u_{r}^{r}=-[R_{r}+j\omega_{r}(L_{r}-\frac{L_{m}}{k})]i_{r}^{r}$$

The negative resistance part in Equation (10) is offset by the rotor resistance *R*_r_. The rotor side is equivalent to a pure inductive load, which minimizes the amplitude of the rotor voltage and makes it easiest to meet the rotor voltage constraint. The rotor voltage can also be expressed as:

(12) $$u_{r}^{r}=-\left(1+\frac{\sigma L_{r}L_{s}k}{L_{m}^{2}k-L_{m}L_{s}}\right)e_{r}^{r}$$

To avoid the rotor-side overcurrent and the saturation of RSC, the voltage and current constraints are obtained as follows:

(13) $$\left\{\begin{array}{l} \left|i_{r}^{r}\right|=\left|\frac{k}{L_{m}k-L_{s}}\right|\Psi_{\mathrm{sm}}\leq 2I_{r}\\ \left|u_{r}^{r}\right|=\left|1+\frac{\sigma L_{r}L_{s}k}{L_{m}^{2}k-L_{m}L_{s}}\right|E_{\mathrm{rm}}\leq U_{\mathrm{bus}} \end{array}\right.$$

Among them, *I*_r_ is the rated value of the rotor current, *U*_bus_ is the rated voltage of the DC bus, *Ψ*_sm_ is the peak value of the stator flux during the fault, and *E*_rm_ is the peak value of the EMF during the fault. The value range of *k* can be obtained:

(14) 0.34≤k≤0.78

**Figure 3:**
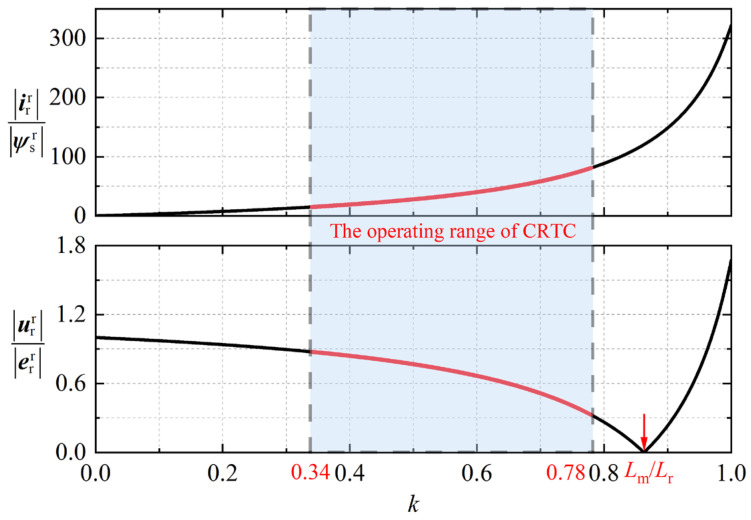
Rotor current and voltage amplitude curves.

The curves of rotor current and rotor voltage under different values of *k* are shown in Figure 3. Within the range of voltage and current constraints, the rotor current is positively correlated with *k*, and the rotor voltage is negatively correlated with *k*.

To suppress the rotor overcurrent while minimizing the risk of RSC oversaturation, the CRTC still needs an optimal value *k*_opt_. Therefore, this paper adopts PSO algorithm to obtain *k*_opt_. When the maximum value of the rotor current and the maximum value of the rotor voltage are closest to the rated value, the tracking coefficient *k* can be regarded as the optimal solution. The fitness is designed as:

(15) $$f(k)={\left(\left|i_{r}^{r}\right|-I_{r}\right)}^{2}+{\left(\left|u_{r}^{r}\right|-U_{r}\right)}^{2}$$

where *U*_r_ is the rated value of the rotor voltage.

In this method, the number of particles *N* is 100, and the maximum number of iterations *N*_max_ is 50. The velocity and new position of particles are updated by the following equation:

(16) $$\left\{\begin{array}{l} v_{ij}(t+1)=c_{1}r_{1}(t)[p_{b}-k_{ij}(t)]+c_{2}r_{2}(t)[g_{b}-k_{ij}(t)]+wv_{ij}(t)\\ k_{ij}(t+1)=k_{ij}(t)+v_{ij}(t+1) \end{array}\right.$$

**Figure 4:**
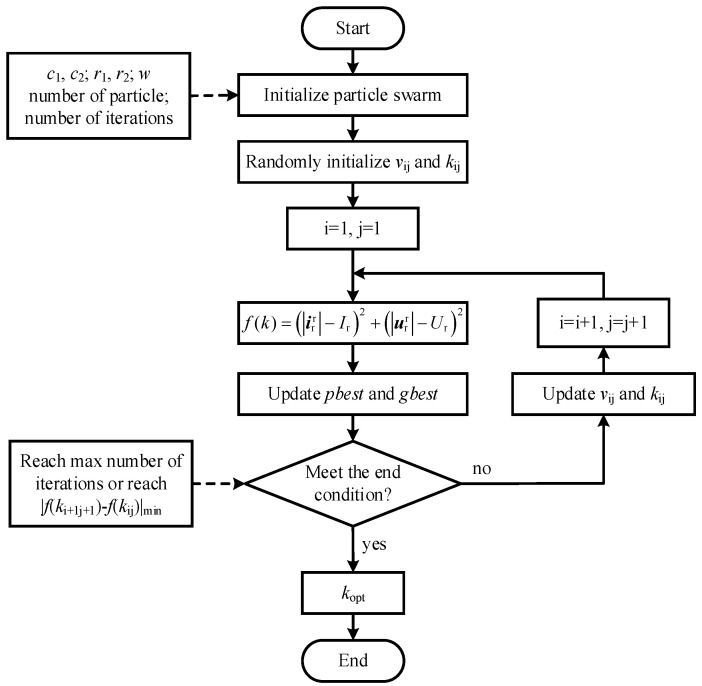
PSO algorithm flow chart.

Among them, *v*_ij_ is the velocity of particles; *k*_ij_ is the current position of particles; *c*_1_ and *c*_2_ are learning factors, *c*_1_ = 1.5, *c*_2_ = 1.5; *r*_1_, *r*_2_ are random numbers between (0, 1), *r*_1_ = *r*_2_ = 0.5; and *w* is the inertia factor, *w* = 0.9 − 0.5 × (*n*/*N*_max_), (*n* < *N*_max_). In each iteration, the particles update themselves by tracking (*p_b_*, *g*_b_); *p*_b_ is the local optimal value; *g*_b_ is the global optimal value. The optimization is stopped when the improvement of the global best solution is less than $10^{-4}$ over 10 consecutive iterations. Finally, *k*_opt_ = 0.53 is obtained by the algorithm. The flow chart of PSO algorithm is shown in Figure 4.

**Figure 5:**
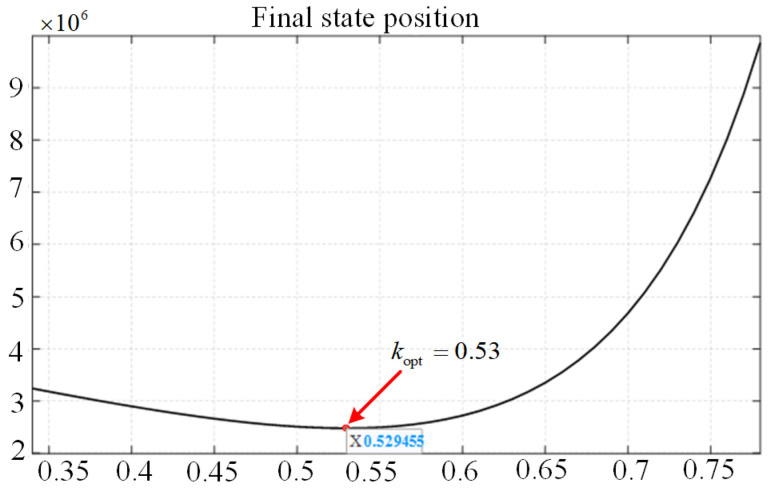
The final state position diagram of PSO algorithm.

Firstly, the population k is initialized, the fitness of each particle is evaluated, and the currents *p*_best_ and *g*_best_ are obtained. Secondly, according to Equation (15), the velocity and position of particles are updated. Thirdly, the fitness values of every new position are calculated and compared; the *p*_best_ and *g*_best_ are updated. Then, if the maximum number of iterations or the minimum difference between two adjacent fitness values is reached, *k*_opt_ is obtained. Otherwise, the velocity and position of the particles are re-updated. Finally, *k*_opt_ = 0.53 is obtained by the algorithm, and the final state position of the PSO algorithm is shown in Figure 5.

### 3.2. Improved Crowbar

**Figure 6:**
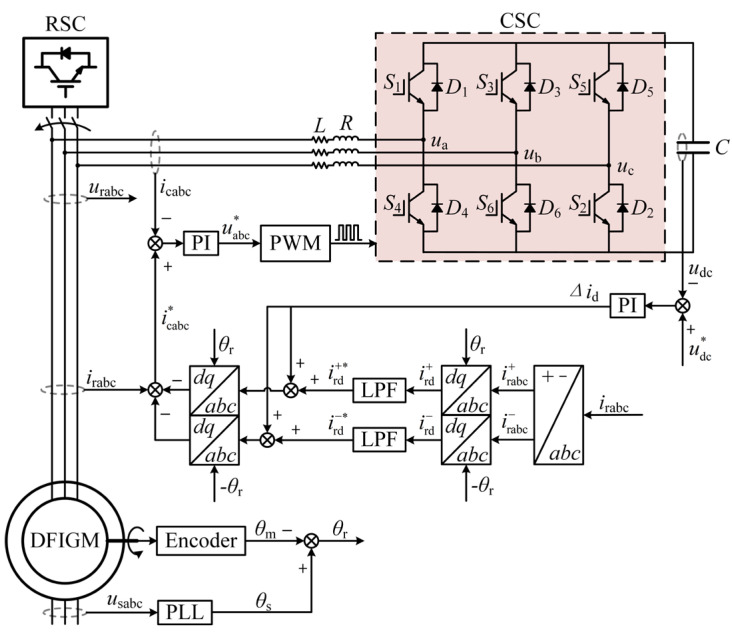
DFIGM system with improved crowbar.

If the dip depth is larger than 50%, it is difficult to meet the LVRT requirements by software scheme. This paper proposes an improved crowbar to achieve LVRT, as shown in Figure 6.

Among them, i_cabc_ is the output current of the AC side of the improved crowbar; *u*_a_, *u*_b_, and *u*_c_ are the three-phase AC voltages of CSC; *S*_1_–*S*_6_ represent six IGBT switches; *D*_1_–*D*_6_ represent six freewheeling diodes; *u*_dc_ is the capacitor voltage at both ends of the DC side.

The *i*_p_-*i*_q_ detection method is adopted for current detection. To obtain the rotor active current $i_{\mathrm{rd}}^{*}$ with only the fundamental component, the positive and negative-sequence components of the rotor active current *i*_rd_ are processed by low-pass filtering (LPF). The detected current subtracts the active current with the fundamental component; the compensation current $i_{c}^{*}$ can be obtained, which is composed of the harmonic component and the reactive component. To further enhance the compensation characteristics, a single closed-loop PI control method is adopted to keep the DC side voltage stable. The active current command signal Δ*i*_d_ is superimposed on the fundamental wave $i_{\mathrm{rd}}^{*}$ of the active current, so that the DC side capacitor of the CSC can exchange active and reactive power with the DFIGM.

**Figure 7:**
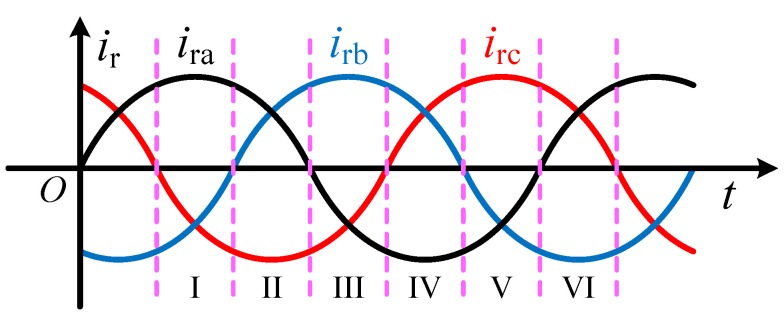
Definition of rotor current sector.

At any time after the improved crowbar branch is operated, the flow of current inside the improved crowbar is determined. According to the alternating characteristics of the three-phase current, the rotor current can be divided into six sectors in a period, as shown in Figure 7. According to the operation process of the improved crowbar, CSC is mainly divided into rectification mode and inversion mode. 

**Figure 8:**
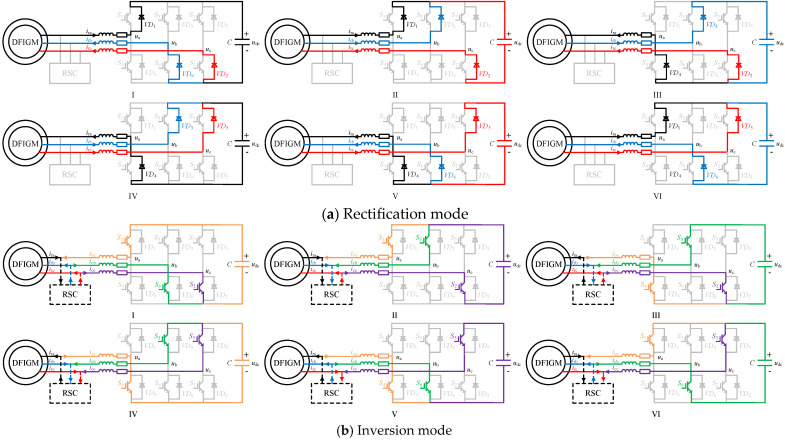
Current flow inside the improved crowbar branch.

Based on the rotor current sector, the internal current flow of the improved crowbar in these two operating modes is shown in Figure 8. When the improved crowbar absorbs the fault energy, the RSC is blocked and the CSC operates in the rectification mode. On the basis of the rotor current sector, the rectification mode is divided into six working states, as shown in Figure 8a. In the rectification mode, the IGBT in the CSC does not work; the rotor current flows through the DC side capacitor by the diode. Taking the state I as an example, which corresponds to the sector I of the rotor current, *i*_ra_ flows into the CSC to the capacitor through D1; *i*_rb_ and *i*_rc_ flow out of the CSC from the capacitor through *D*_6_ and *D*_2_, respectively. CSC operates in the inversion mode when the improved crowbar performs reactive power compensation or eliminates harmonics. Similarly, the inversion mode is also divided into six working states, as shown in Figure 8b. In inversion mode, the diode in CSC does not work; the DC side current is transmitted to the rotor side through the IGBT. Taking the state I as an example, which corresponds to the sector I of the rotor current, the output current of CSC is opposite to the harmonic polarity of the rotor current. *i*_ca_ flows out of the CSC from the capacitor through *S*_1_; *i*_cb_ and *i*_cc_ flow into the CSC to the capacitor through *S*_6_ and *S*_2,_ respectively.

According to KVL, the state equation of the improved crowbar is:

(17) $$\left\{\begin{array}{l} L\frac{di_{\mathrm{ca}}}{dt}=u_{\mathrm{ra}}-Ri_{\mathrm{ca}}-(m_{a}-\frac{1}{3}\sum_{i=a,b,c}m_{i})u_{\mathrm{dc}}\\ L\frac{di_{\mathrm{cb}}}{dt}=u_{\mathrm{rb}}-Ri_{\mathrm{cb}}-(m_{b}-\frac{1}{3}\sum_{i=a,b,c}m_{i})u_{\mathrm{dc}}\\ L\frac{di_{\mathrm{cc}}}{dt}=u_{\mathrm{rc}}-Ri_{\mathrm{cc}}-(m_{c}-\frac{1}{3}\sum_{i=a,b,c}m_{i})u_{\mathrm{dc}}\\ C\frac{du_{\mathrm{dc}}}{dt}=m_{a}u_{\mathrm{ra}}+m_{b}u_{\mathrm{rb}}+m_{c}u_{\mathrm{rc}} \end{array}\right.$$

where *m*_i_ (i = a, b, c) is the switching function; *m*_i_ = 1 when the upper bridge arm is turned on and the lower bridge arm is turned off; when the upper bridge arm is turned off and the lower bridge arm is turned on, *m*_i_ = 0.

The mathematical model of the improved crowbar in the dq coordinate system is:

(18) $$\left\{\begin{array}{l} L\frac{di_{\mathrm{cd}}}{dt}=L\omega_{r}i_{\mathrm{cq}}+u_{\mathrm{rd}}-Ri_{\mathrm{cd}}-u_{\mathrm{cd}}\\ L\frac{di_{\mathrm{cq}}}{dt}=-L\omega_{r}i_{d}+u_{\mathrm{rq}}-Ri_{\mathrm{cq}}-u_{\mathrm{cq}}\\ C\frac{du_{\mathrm{dc}}}{dt}=m_{d}i_{\mathrm{cd}}+m_{q}i_{\mathrm{cq}} \end{array}\right.$$

Here, *m*_d_ and *m*_q_ are the switching functions obtained by the transformation of *m*_a_, *m*_b_, and *m*_c_. When the improved crowbar is put into operation, the improved crowbar increases the rotor circuit impedance, which can suppress the rotor circuit current. The EMF and rotor voltage at this time can be expressed as:

(19) $$\left\{\begin{array}{l} e_{r}^{r}=-\left[j\omega_{r}(\sigma L_{r}+L-\frac{1}{\omega_{r}^{2}C})+R_{r}+R\right]i_{r}^{r}\\ u_{r}^{r}=-\left[j\omega_{r}(L-\frac{1}{\omega_{r}^{2}C})+R\right]i_{r}^{r} \end{array}\right.$$

**Figure 9:**
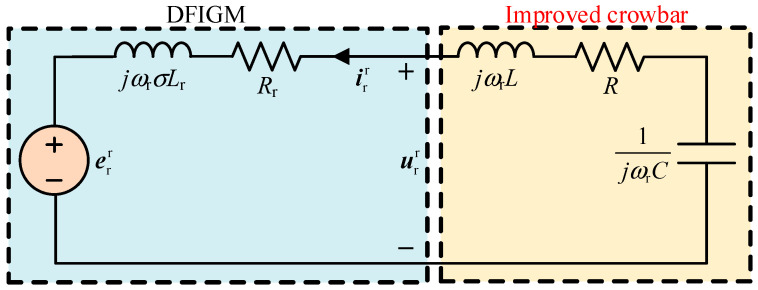
The equivalent circuit with improved crowbar.

The equivalent circuit of the rotor side is shown in Figure 9. Regardless of the value of *R*, *L*, and *C*, the rotor voltage is smaller than the EMF, which can reduce the voltage requirement of RSC port during the fault.

## 4. Simulation Results

**Figure 10:**
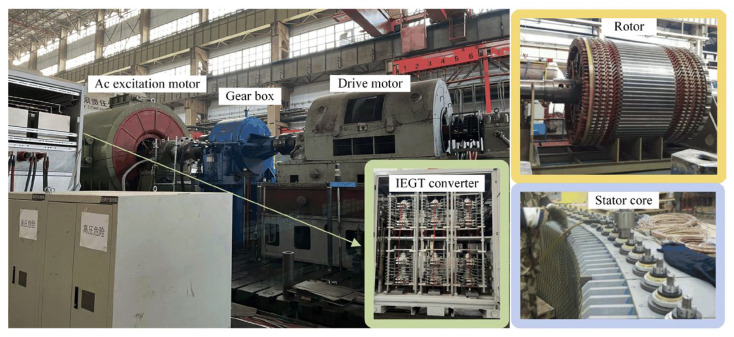
Ten MW DFIGM prototype.

The simulation model of 10 MW DFIGM is established, and its prototype and parameters are shown in Figure 10 and Table 1.

To verify the correctness of the previous analysis and the effectiveness of the proposed control method, 50% symmetrical voltage dip and interphase voltage dip are simulated in this paper below.

### 4.1. Analysis of EMF

**Figure 11:**
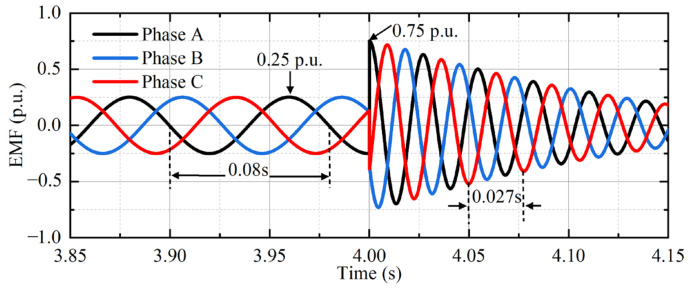
EMF simulation waveform under symmetrical dip.

Take the symmetrical complete dip as an example, that is, *d* = 1 and *s* = 0.25. The waveform of EMF is shown in Figure 11.

Under normal operating conditions, the amplitude of EMF is 0.25 p.u. At the beginning of the fault, the EMF amplitude is nearly three times that of the normal condition, which has a strong impact on the RSC.

**Figure 12:**
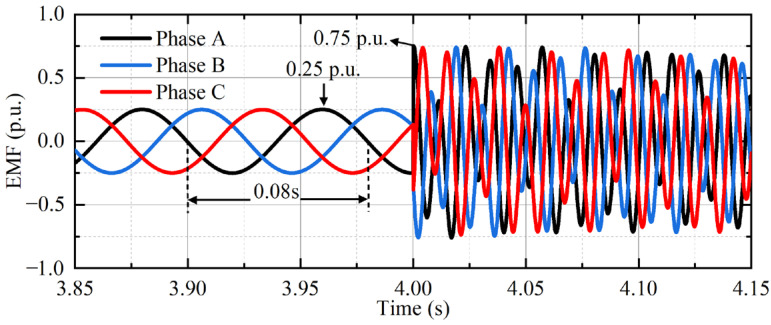
EMF simulation waveform under asymmetrical dip.

**Figure 13:**
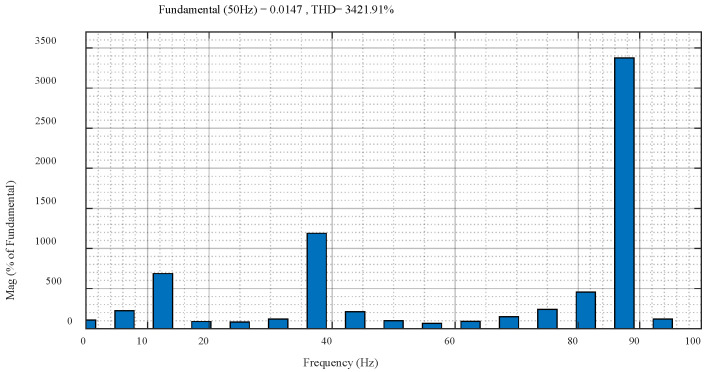
FFT analysis of EMF waveform.

Take the interphase complete dip as an example, that is, also, *d* = 1 and *s* = 0.25. The waveform of EMF is shown in Figure 12. At the beginning of the fault, the EMF amplitude is nearly three times that of the normal condition. The harmonic frequencies of EMF are mainly concentrated in the vicinity of 12.5 Hz, 37.5 Hz, and 87.5 Hz, as shown in Figure 13.

### 4.2. Fifty % Symmetrical Voltage Dip

**Figure 14:**
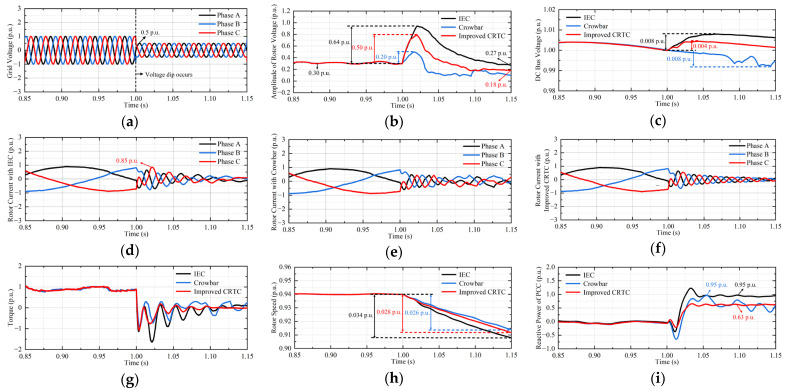
Waveforms of DFIGM parameters. (**a**) Gird voltage. (**b**) Amplitude of stator voltage. (**c**) DC bus voltage. (**d**) Rotor current with IEC. (**e**) Rotor current with crowbar. (**f**) Rotor current with improved CRTC. (**g**) Electromagnetic torque. (**h**) Rotor speed. (**i**) Reactive power at PCC.

This paper takes the pumping mode as an example for analysis, the three methods of IEC, crowbar, and LVRT method proposed in this paper are compared. From Figure 14a, the voltage amplitude of the power grid dropped to 0.5 p.u. at 1 s and returned to normal after 0.15 s. In this case, the improved CRTC is put into operation.

From Figure 14, the improved CRTC is superior to crowbar in improving the LVRT ability of the DFIGM without additional auxiliary devices. In terms of rotor voltage amplitude, IEC has the worst suppression effect; its increase in amplitude is about 1.2 times that of the improved CRTC. For software scheme, improved CRTC can reduce the oversaturation of RSC. Compared with the other two methods, the improved CRTC makes the DC bus voltage ripple minimal and more stable. Three methods all effectively suppress the rotor overcurrent. In terms of electromagnetic torque, the improved CRTC makes the electromagnetic torque almost no pulsation in the later stage of the fault. In terms of speed, three methods all reduce the response of the DFIGM to the rotor speed control command. As a result, the rotor speed changes drastically during the fault. Compared with IEC, the improved CRTC reduces the fluctuation of rotor speed by 17.6%. Three methods all weaken the control effect of RSC on the stator reactive power, the reactive power absorbed in IEC is the most, which is about 1.5 times that of improved CRTC. Considering the improvement of unit performance, the improved CRTC is the best choice.

### 4.3. Interphase Voltage Dip

**Figure 15:**
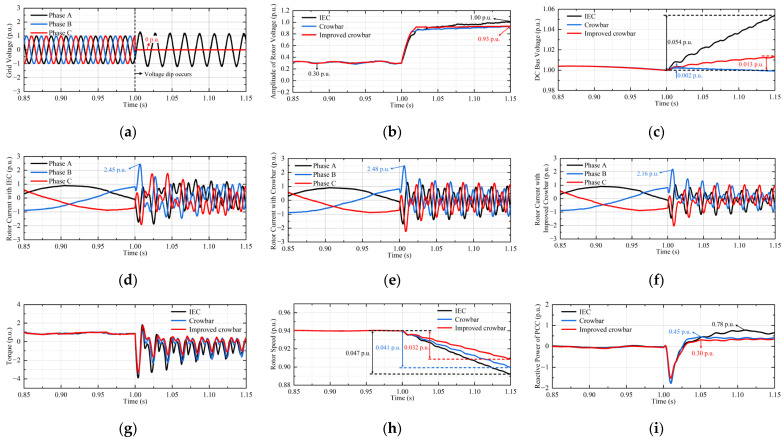
Waveforms of DFIGM parameters. (**a**) Gird voltage. (**b**) Amplitude of stator voltage. (**c**) DC bus voltage. (**d**) Rotor current with IEC. (**e**) Rotor current with crowbar. (**f**) Rotor current with improved crowbar. (**g**) Electromagnetic torque. (**h**) Rotor speed. (**i**) Reactive power at PCC.

The interphase voltage dip is shown in Figure 15a; the short circuit occurs between the grid voltages of phase B and phase C at 1 s, and it returned to normal after 0.15 s. In this case, the improved crowbar is put into operation.

From Figure 15, the improved crowbar can more effectively achieve LVRT. Due to the limitation of RSC capacity, IEC has the worst effect in improving the LVRT capability. Due to the limitation of the crowbar resistance, crowbar cannot take into account the control effect of the rotor current and the DC bus voltage. Although the DC bus voltage of crowbar fluctuates the least, improved crowbar has a better effect on suppressing the rotor overcurrent. Both the electromagnetic torque ripple and speed fluctuation with improved crowbar are the smallest, the attenuation is about 75% of that of crowbar. Improved crowbar minimizes the reactive power absorbed by DFIGM.

### 4.4. Power Quality Analysis of Grid

**Figure 16:**
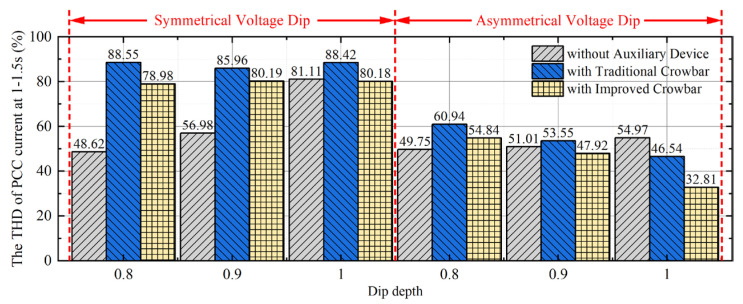
The THD of the PCC current.

In the simulation, FFT analysis of PCC current is carried out to observe the influence of crowbar on the harmonic content of grid current. Taking the current of phase A with a reference frequency of 50 Hz as an example, the THD of the PCC current from the fault to the recovery (1 s–1.5 s) is shown in Figure 16. The THD values are from deterministic single-run simulations; the results are deterministic.

Under 80% symmetrical voltage dip, the THD of PCC current is increased by nearly one time after adopting the traditional crowbar. It shows that the traditional crowbar will reduce the power quality under the fault with low asymmetry. Compared with the traditional crowbar, the improved crowbar can reduce the harmonic content of the PCC current by nearly 15%. Under asymmetrical voltage dip, the greater the asymmetry, the more obvious the effect of the improved crowbar on improving the power quality. For the interphase voltage dip, the THD with the improved crowbar is the lowest, which is 75.5% of the traditional crowbar and 59.7% of no devices. Therefore, the improved crowbar can improve the power quality of the grid.

## 5. Experiment Results

**Figure 17:**
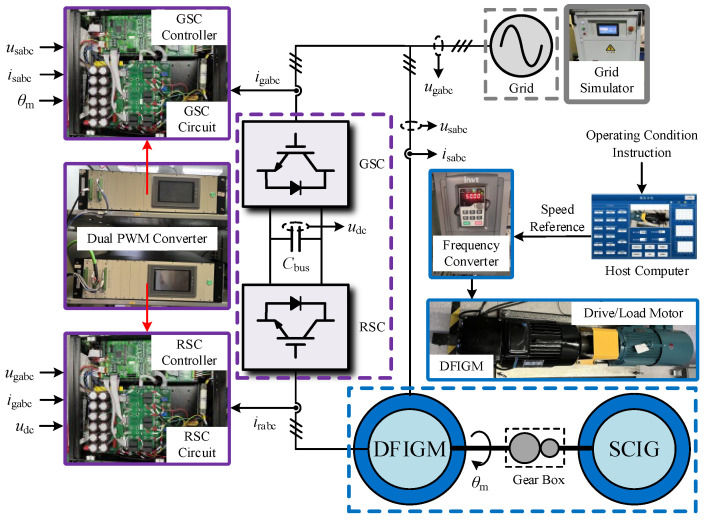
Experimental platform of a 5.5 kW DFIGM system.

According to the principle of proportional reduction, the 5.5 kW experimental platform is established in Figure 17, which is adapted to verify the effectiveness of the improved CRTC. The steady-state before the fault is set to DFIGM running in the generation mode. Before the fault, the traditional vector control strategy was adopted in the dual PWM converter.

**Figure 18:**
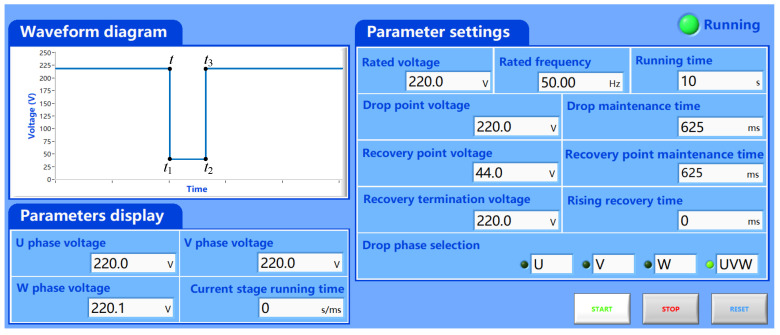
Grid simulator settings.

The parameter configuration of the grid simulator is shown in Figure 18; the dip depth is 0.8, the grid simulator is configured with a rated frequency of 50 Hz, the voltage at the dip point is set to 220 V, and the fault time lasts 625 ms. The stator-side output active power is 2 kW, the reactive power is 0 Var, and the rotor speed is 1800 r/min.

**Figure 19:**
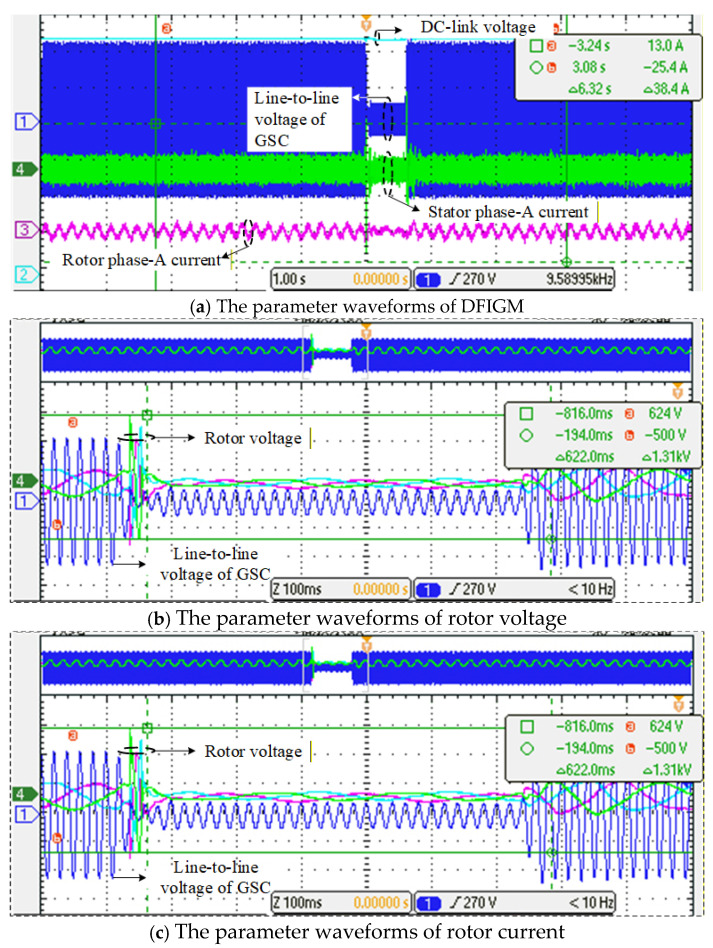

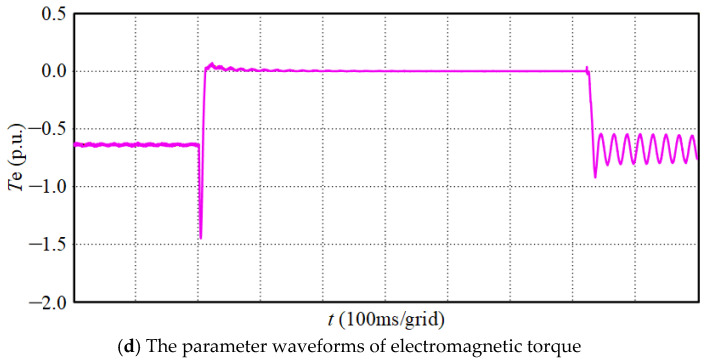
The waveforms under 80% symmetrical dip of grid voltage.

During the fault, the improved CRTC was adopted in the RSC. The experimental waveforms are shown in Figure 19. As shown in Figure 19a, the DC-link voltage of the generator remains stable during the fault, indicating that the improved CRTC can effectively suppress DC-link voltage fluctuations. From Figure 19b, it can be observed that with the improved CRTC, the impedance of RSC is equivalently shaped into a purely inductive form, and the rotor current effectively restrains the variation in magnetic flux. The peak rotor voltage during the fault is limited to 624 V, which is lower than the rated DC-link voltage of 650 V. Therefore, the improved CRTC satisfies the DC-link voltage safety constraint and effectively prevents the freewheeling diodes in the RSC from conducting during the fault, thereby ensuring the controllability of the RSC.

As shown in Figure 19c, the peak rotor current is 5.80 A, twice below the rated rotor current, as well as the relay protection threshold. The improved CRTC can effectively suppress rotor overcurrent during fault conditions, preventing overcurrent-induced damage to the RSC and achieving the objective of maintaining grid connection during voltage dips. Since the internal resistance of the kW-scale prototype is larger than that of the MW-scale system, the corresponding flux decay coefficient in the experimental setup is higher than that in the simulation model, resulting in a faster decay rate of the induced electromotive force (EMF). Both the rotor current and rotor voltage in the experiments stabilize rapidly after experiencing a short period of large transient fluctuations.

The electromagnetic torque waveform is shown in Figure 19d. Affected by the variation in rotor current, the electromagnetic torque undergoes a brief oscillation during the fault and then remains approximately at zero, which is consistent with the theoretical analysis presented earlier. This demonstrates that the improved CRTC is capable of effectively eliminating electromagnetic torque pulsations.

**Figure 20:**
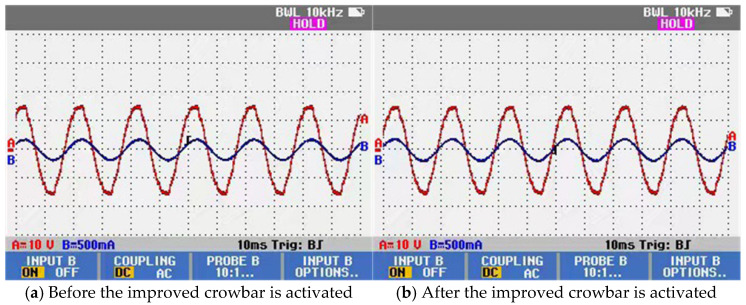
Voltage and current waveforms of the main circuit.

The reactive power compensation capability is experimentally verified. The A-phase voltage and current of the main circuit are used as an example. As shown in Figure 20a, a noticeable phase difference exists between the voltage and current waveforms. After the improved crowbar is activated, the voltage and current of the main circuit become in phase, resulting in a unity power factor, as illustrated in Figure 20b. These results demonstrate that the improved crowbar provides effective reactive power compensation for the main circuit.

**Figure 21:**
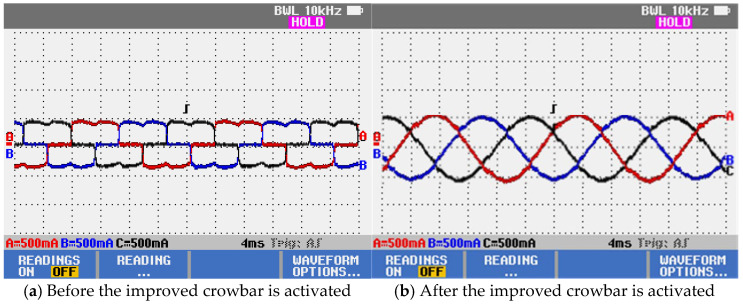
Current waveforms of the main circuit.

To verify the effectiveness of the improved crowbar in mitigating current harmonics, the three-phase AC motor is replaced with a nonlinear inductive-resistive load based on an uncontrolled rectifier. The main-circuit current waveforms before and after the activation of the improved crowbar are shown in Figure 21. The comparison indicates that, after the improved crowbar branch is activated, the harmonic content of the main-circuit current is significantly reduced.

**Figure 22:**
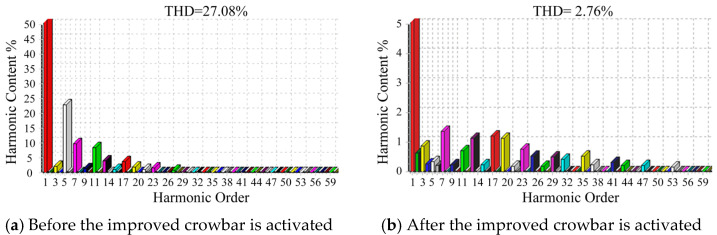
Spectrum diagrams of A-phase current in main circuit.

To more clearly illustrate the performance of the improved crowbar, the total harmonic distortion (THD) of the A-phase current in the main circuit is measured using a Fluke 434 power quality analyzer, and the experimental results are presented in Figure 22.

Due to the presence of the nonlinear inductive-resistive load, the main-circuit current waveform becomes distorted and contains substantial harmonic components, with the THD of the A-phase current reaching 27.08%. After the improved crowbar is activated, the main-circuit current waveform approaches a sinusoidal shape, and the THD of the A-phase current is reduced to 2.76%. These results demonstrate that the improved crowbar effectively mitigates the harmonic content of the main-circuit current.

## 6. Conclusions

In this paper, to solve the problem of variable-speed pumped storage power generator-motor under voltage dip fault, a cooperative control strategy based on improved CRTC and improved active crowbar is proposed. The proposed scheme can effectively suppress the rotor overcurrent; it makes the DC bus voltage more stable; the electromagnetic torque oscillates less during the fault. From the perspective of power quality, the improved crowbar can suppress the increase in the negative-sequence component of the PCC current during the fault, eliminate the harmonic content of the grid current, and reduce the THD of the PCC current. The control strategy proposed in this paper makes up for the shortcomings of the traditional single auxiliary device, it can improve the LVRT performance and improve the power quality at the same time.

Owing to the impracticality of full-scale fault testing for a 10-MW machine, this study adopts a combined simulation–experiment validation framework, while future work will extend the verification to higher power levels with a focus on thermal stress, device reliability, and long-term robustness under repeated grid faults.

## Figures and Tables

**Table 1:** Parameter of 10 MW DFIGM.

Symbol	Parameter	Value
*P*_s_	Rated stator power	10 MW
*U*_s_	Rated stator voltage	10.5 kV
*I*_s_	Rated stator current	611 A
*f*_s_	Rated stator frequency	50 Hz
*U*_bus_	Rated DC-link voltage	5000 V
*P*	Pole pair	6
*s*	Turn ratio (*N*_s_/*N*_r_)	0.5409
*R*_s_	Stator resistance	0.0532 Ω
*R*_r_	Rotor resistance	0.0261 Ω
*L*_m_	Magnetizing inductance	29.6 mH
*L*_sσ_	Stator leakage inductance	3.1 mH
*L*_rσ_	Rotor leakage inductance	5.9 mH

## Data Availability

The original contributions presented in this study are included in the article. Further inquiries can be directed to the corresponding authors.
